# Advancements of Data Anomaly Detection Research in Wireless Sensor Networks: A Survey and Open Issues

**DOI:** 10.3390/s130810087

**Published:** 2013-08-07

**Authors:** Murad A. Rassam, Anazida Zainal, Mohd Aizaini Maarof

**Affiliations:** 1 Faculty of Computing, Universiti Teknologi Malaysia, Johor 81310, Malaysia; E-Mail: aizaini@utm.my; 2 Faculty of Engineering and Information Technology, Taiz University, Taiz 6803, Yemen

**Keywords:** wireless sensor networks (WSNs), data anomaly detection, detection effectiveness, detection efficiency, energy consumption

## Abstract

Wireless Sensor Networks (WSNs) are important and necessary platforms for the future as the concept “*Internet of Things*” has emerged lately. They are used for monitoring, tracking, or controlling of many applications in industry, health care, habitat, and military. However, the quality of data collected by sensor nodes is affected by anomalies that occur due to various reasons, such as node failures, reading errors, unusual events, and malicious attacks. Therefore, anomaly detection is a necessary process to ensure the quality of sensor data before it is utilized for making decisions. In this review, we present the challenges of anomaly detection in WSNs and state the requirements to design efficient and effective anomaly detection models. We then review the latest advancements of data anomaly detection research in WSNs and classify current detection approaches in five main classes based on the detection methods used to design these approaches. Varieties of the state-of-the-art models for each class are covered and their limitations are highlighted to provide ideas for potential future works. Furthermore, the reviewed approaches are compared and evaluated based on how well they meet the stated requirements. Finally, the general limitations of current approaches are mentioned and further research opportunities are suggested and discussed.

## 1. Introduction

Wireless sensor networks (WSNs) are networks of tiny, low cost, low energy, and multifunctional sensors which are densely deployed to monitor a phenomenon, track an object, or control a process [1]. WSNs are used in many application domains which include: personal applications such as home automation; business applications such as sales tracking; industrial applications such as architectural and control; and military applications such as enemy target monitoring and tracking [1–3]. A new concept that seems to be the future of WSNs is the Internet of Things (IoT) which expects that each object in human life will be equipped with sensors that communicate with each other to constitute a network that make life much easier [4]. In the IoT, sensor nodes join the internet dynamically, and use internet infrastructure to collaborate and perform their tasks [5]. According to [6], the future internet as known by IoT is expected to be a “world-wide network of interconnected objects uniquely addressable, based on standard communication protocols”. One of the most important elements in the IoT paradigm according to [7] is the WSNs because they act as a digital skin that provide a medium to access information about the physical world by any computational system. Different technologies are developed to make the integration of WSNs with IoT such as the 6LowPAN standard defined by IETF [8] that allows the transmission of IPv6 packets in computational restricted networks.

WSNs have been approached from different disciplines including networking, embedded systems, information processing, distributed systems and signal processing. As a result, a variety of research areas have been evolved, including routing protocols, localization strategies, sensing hardware design, query processing, data mining, information processing, security, and privacy.

Sensor data analysis is of high importance to decision makers. It was reported by [9] that the purpose of using a WSN is not only to collect data from the field of deployment, but more importantly the analysis of this data at timely manner that allows for making some important decisions. Therefore, the data quality is the main concern since it reflects the true world state of WSN applications. Unfortunately, the raw measurements collected by sensor nodes, especially from large scale WSNs, often suffer from inaccuracy and incompleteness [10]. These inaccurate sensor measurements may be produced due to reasons related to sensor device itself or the sensing environment. Resource constraints of sensor devices in terms of storage, energy, processing, and bandwidth may contribute to node failures and therefore reporting of anomalous readings. Other reasons that are related to the environment include the harshness and the difficulties of the deployment area may also result in erroneous data [11–13]. In addition, malicious attacks such as denial of service, sinkhole, black hole, selective forwarding, and wormhole attacks [3,14–20] may also contribute to generating such inaccurate and low quality data. Besides, physical interruptions such as destruction or movements of sensor devices caused by humans or animals may affect the data collection process and lead to anomalous measurements [1].

The inaccurate or incomplete data measurements caused by the aforementioned reasons are known as anomalies. An anomaly is defined in [21] as an observation that seems to be inconsistent with the rest of a dataset. Anomaly detection is defined in [22] as a process of finding data patterns that deviate from expected behavior. The anomaly detection problem has been studied from different perspectives such as data security, data mining, or pattern recognition. The term “anomaly” is variously known in the literature as an outlier, fault or deviation.

Many varieties of anomaly detection solutions exist for traditional (wired) networks depending on the specific domain they are used for. However, these solutions cannot be ported directly for WSNs because of the limitations of these networks in energy, processing, bandwidth, and storage capacity. Moreover, the anomaly detection techniques for traditional networks focus on the network layer itself, while this survey is more concerned with the data on the application layer of WSN. Therefore, a modification of these techniques or a design of new suitable techniques especially for WSNs is required [18,23,24].

Anomaly detection solutions in WSNs are characterized by their detection effectiveness and their efficiency in utilizing the limited network resources [9]. Detection effectiveness is represented by detection accuracy, detection rate, and false alarms. Detection efficiency is represented by energy consumption and memory utilization. Therefore, any proposed solution for anomaly detection should consider the improvement of detection effectiveness while consuming less energy and storage during detection process.

**Figure 1.**
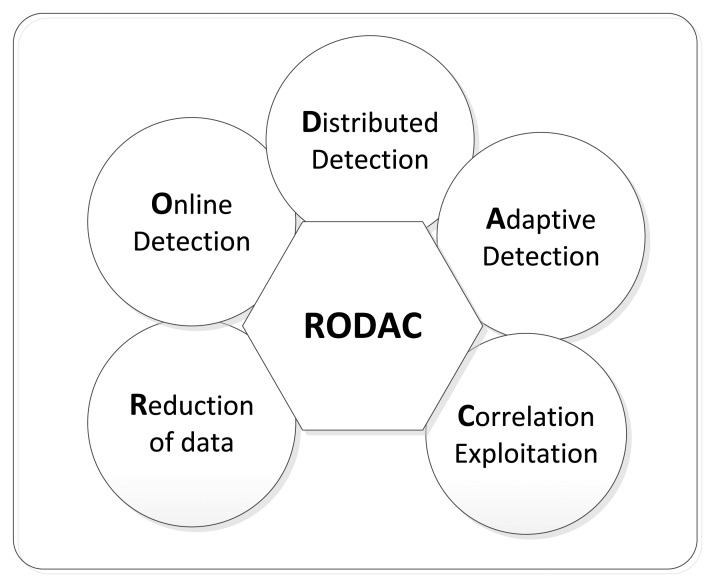
Requirements of Anomaly Detection in WSNs (RODAC).

In this survey, the challenges that face the design and development of efficient and effective anomaly detection models in WSN are presented. These challenges are inherited from the characteristics of sensor nodes themselves in terms of resource restrictions that make the direct utilization of existing anomaly detection models from other platforms impossible. Knowing the challenges guides this survey to formulate the requirements to design efficient and effective anomaly detection models that overcome these challenges. These requirements are abbreviated as RODAC, which includes five items that are shown in Figure 1.

As shown in Figure 1, five main requirements are necessary to design effective and efficient anomaly detection models which include the need for dimension reduction, online detection, distributed detection, adaptive detection and data correlation exploitation. In particular, the online and distributed detection are among the most important requirements for the design of efficient and effective anomaly detection solutions. Online detection ensures that real time anomalies are not missed while distributed detection ensures that the limited resources are efficiently utilized by spreading the computational load over the network. However, the other requirements are strongly related to those two requirements. For example, data reduction that aims at reducing the dimension of data to enhance the efficiency exploits the feature correlation in a distributed structure. Adaptive detection is necessary for real time detection in dynamic environments where the changes in data distribution affect the detection effectiveness. Correlation in sensor data of close neighborhoods was found to enhance the detection effectiveness by the mean of distributed detection in close neighborhoods. Similar components of RODAC requirements such as distributed processing and data correlation exploitation have been considered to design an efficient data collection approaches in sensor networks [25,26]. In [26], a statistics-based data collection model was proposed by introducing approximations with probabilistic confidences. Meanwhile, in [25], another predictive-based data collection model for sensor networks was designed by approximating the joint probability distribution over the sensors using undirected graphical models in order to exploit both the spatial correlations and the broadcast transmission. It was claimed that both models are significantly more computationally efficient in terms of both time and energy.

In addition, the existing models are classified based on the methods used to design the detection models into: statistical-based, clustering-based, classification-based, nearest neighbor-based and others anomaly detection models.

The aim of this survey is to help readers better understand the RODAC requirements and determine the potential improvements on the existing anomaly detection models based on them. Furthermore, it aims to introduce guidelines for designing new anomaly detection models that consider the RODAC requirements so that detection efficiency and effectiveness are guaranteed. A comparison of existing detection models from each class is conducted based on how well they meet the RODAC requirements and the limitations of each model are mentioned. Additionally, the general limitations of current approaches are presented to provide potential opportunities for further research work. To the best of our knowledge, there is no recent survey that addresses the problem of anomaly detection in WSNs based on the same set of RODAC requirements.

The rest of this survey is structured as follows: related review works are discussed and the significance of our survey compared to existing survey works is further highlighted in Section 2. Section 3 provides a detailed background and preliminaries about anomaly detection and related issues in WSNs. In Section 4, the current anomaly detection models are classified into five classes. Furthermore, the state-of-the-art models are described and their limitations are highlighted for each class in Section 4. The analysis and evaluation of the current models based on their satisfaction to the RODAC requirements is discussed in Section 5. Section 6 highlights the limitations of the current anomaly detection models and suggests further opportunities for future research. Section 7 concludes this survey.

## 2. Related Works

The importance of anomaly detection for ensuring sensor data quality and detecting malicious attacks that affect network functionality and data integrity has encouraged efforts of some previous studies to survey WSN security and anomaly detection models. This section points out the current existing surveys of anomaly detection in WSN and gives the differences that distinguish this survey from them.

A technique-based classification of anomaly detection models in WSN was proposed by Rajasegarar and co-authors in [27,28]. In both surveys, anomaly detection models were categorized into statistical models and non-parametric models based on the techniques used to develop the detection model. Non-parametric models were further categorized into rule-based, CUSUM-based, data-clustering based, density-based, and support vector machines (SVM)-based models. In statistical-based models, the underlying density distribution of data types (normal or anomalous) is either known *a priori* or estimated using density estimation techniques. On the contrary, non-parametric models do not assume any prior knowledge about data types and use different measures to capture the normal data behavior to be matched against the subsequent measurement behavior.

Another technique-based comprehensive classification of outlier detection models was introduced in [9]. In this classification, the non-parametric models were considered as a part of statistical models and two additional classes were added, which are the nearest neighbor-based and spectral-decomposition-based models. SVM-based models were classified among classification-based models in addition to the Bayesian network models. Bayesian networks models were further categorized into three sub-categories, which are the Naive Bayesian, Belief Bayesian, and Dynamic Bayesian models.

A technical taxonomy of anomaly detection models in WSN was presented in [29] and focused on three criteria which are the speed of detection, the generality of detection, and the balance between both of them. The taxonomy categorized intrusion detection models according to the network structure into flat-based and hierarchical-based. This taxonomy focused more on the security aspects side of the anomaly as it surveys many intrusion detection models. The taxonomy suggested that rule based models are very fast and suitable for the flat structure WSNs whereas the statistical models are fast for hierarchical structure. In terms of generality, data mining or computational intelligence models were suggested to be the best choice for both structures of WSNs. Furthermore, it was suggested that statistical techniques can achieve a balanced performance between speed and generality in both flat and hierarchical structures of WSNs.

Another survey of anomaly detection in WSN introduced [30] considers different criteria to analyze the current anomaly detection models. It focuses on the types of anomalies based on their scope as data anomalies, node anomalies and network anomalies. The symptoms, scope, detection metric, localization metric and diagnostic of these anomalies are then discussed. In terms of detection strategies, the existing models were discussed based on their method concept, implementation status as tool or algorithm, anomaly type as data, node or network, architecture as centralized or distributed, and the usability of the detection model in terms of the interaction with the user. The similarity between this survey and our proposed survey is in the focus on network structure. However, very few detection models were reviewed and analyzed with this regard in [30].

Different types of sensor network data faults were reviewed and systematically modeled in [31]. Based on the faults modeling, some common features were developed that are helpful in detecting and diagnosing faults. These features were then used to define the commonly observed faults, and give examples of each of these faults from real life sensor data.

The classifications in [9,27,28] are technique-based, although they have slight differences in the classification. Meanwhile, the classifications in [29,30] focused on different criteria with regards to network structure and types of anomalies. In [29], the survey focused on network architecture and evaluated each detection scheme based on detection speed measured by computational complexity and detection generality measured by detection accuracy. However, the computational complexity is not the only factor that affects detection efficiency. For distributed detection models, the communication overhead is more important and consumes the most energy of sensor networks. Our survey is different as it considers detection efficiency in general which is measured by computational complexity and communication overhead. Furthermore, the scope of this survey in terms of the number of models reviewed is broader than existing surveys in the literature.

## 3. Background and Preliminaries

The fast advancement in communication technologies has introduced cheap, low-power and multifunctional devices which leverage the idea of the sensor [1]. Wireless Sensor Networks (WSNs) can be defined as a kind of networks that is formed by tens to thousands of tiny sensors which are densely deployed in an unattended environment. The most critical constraint on the sensor nodes is the energy consumption, since these nodes have very limited and unchangeable power sources. As a result, this restriction influences the design of WSN protocols or algorithms.

The main function of sensor node is to detect events in the sensor field, perform some simple processing tasks and send the data to some authorized party, such as a network administrator. Based on that, the energy is consumed in three forms: sensing, data processing, and communication operations. The most energy is spent on the communication part which involves the transmission and reception of data among the nodes. According to [32], the amount of energy needed to transmit one bit equals the energy needed to perform thousands of CPU operations inside the sensor. Two unique features that differentiate WSNs from other networks were mentioned in [1]: first, the position of sensor nodes is not determined or engineered beforehand and this allows the random deployment at inaccessible environments and disaster relief operations; this feature poses a challenge for designing a self-organizing algorithms and protocols; second, the distributed data processing; instead of sending raw data to fusion node (base station or cluster head), the nodes can use their processing capabilities to locally carry out some simple required computations and send only the summary that is beneficial for decision making.

### 3.1. Wireless Sensor Networks Application Areas

**Figure 2.**
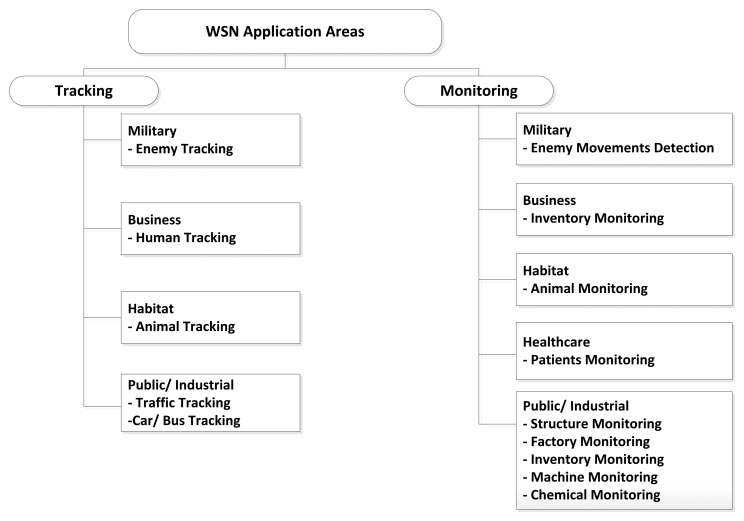
Some of WSN application areas. Adapted from [3].

Sensor networks may be composed of different types of sensors, such as thermal, visual, infrared, and magnetic among others, based on the type of the application they are designed for. This enables WSNs to monitor a variety of conditions that include but are not limited to: temperature, humidity, light, pressure, noise, speed direction of an object, size of an object, and noise levels [33]. The unique features of WSNs ensure that they can be used for a wide range of applications [4]. The authors of [3] categorized WSN applications into two categories: monitoring and tracking. Each category is further categorized into many subcategories. A broad number of monitoring and tracking applications are already implemented and currently in service for public use or industry. However, describing such applications is out of the scope of this survey. Figure 2 shows the classification of WSN application areas with some examples.

### 3.2. Wireless Sensor Networks Structure

**Figure 3.**
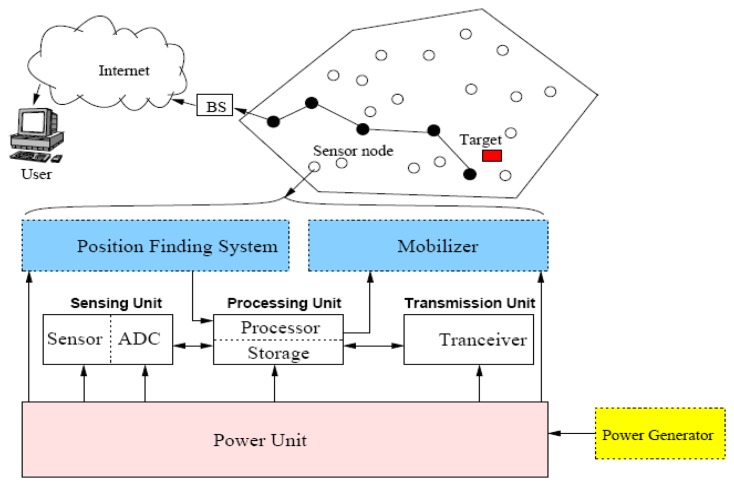
The main sensor node structure [1].

The structure of a WSN is based on the sensor node which is made up of four main components as illustrated in Figure 3. The main components are: sensing unit, data processing unit, power unit, and data transmission unit. The sensing unit is composed of two subunits which are the sensor that collects the data from the field and the analog to digital convertor (ADC) that transfers the analog signal produced by sensor to a digital form to be used by the processing unit. The processing unit uses a small storage media to store necessary data during processing task. The transceiver unit is used for the communication purposes between the node and other nodes or the base station. Three more additional units may be incorporated in the sensor structure depends on the application which are the energy generator that supplies the sensor with additional energy sources, the mobilizer which is sometimes used for supporting the mobility of sensor nodes, and the position finding system that determines the current position of mobile sensor nodes [1].

The structure of a WSN differs by its different layer design strategy from physical layer to network layer. For example, many strategies can be used for source encoding in the physical layer. The designer may choose between the pulse code modulation or delta modulation and the same goes for the channel coding and signal propagation. In the Medium Access Control (MAC) layer, many choices are available for the design of a suitable structure. In the MAC layer, the two main strategies are contention based medium access and contention free medium access. The IEEE 802.15.4 protocol is considered the standard specification for the physical and MAC layers for networks that have low data rates, low energy sources, and short range communications [34].

On the network layer, routing protocols are classified according to network structure into flat-based, hierarchical-based, and location-based [35]. In a flat-based structure, all nodes are treated equally and given the same functionality. In a hierarchical structure, the nodes have different roles in the network according to their responsibilities. In this structure, a cluster head (CH) is given additional responsibilities and resources over other common nodes. In the location-based structure, the position of the nodes is used to determine the most suitable routing path to the target.

### 3.3. Anomaly Detection in Wireless Sensor Networks

Ensuring sensor data quality is crucial for right decision making. Cryptographic and key management techniques are not sufficient to ensure the integrity of data as they cannot protect sensor nodes from insider attacks such as data fabrication. Therefore, anomaly detection models are designed to detect any abnormal behavior in sensor data streams. The following subsections describe the concepts, challenges and requirements of anomaly detection in WSNs.

#### 3.3.1. Definitions and Basic Concepts

Anomalies are defined in [22] as “patterns in data that do not conform to a well-defined notion of normal behavior”. Another definition in [21] as “an observation that appears to be inconsistent with the reminder of a dataset”. Anomalies might appear in data for different reasons, such as malicious activity, for example, cyber-attacks, cards frauds, breakdown of the system or terrorist activity, but the common feature of all of these reasons is that they are of interest to be analyzed [22].

In WSNs, anomalies can be defined as those significant deviations in the sensing data measurements from the normal sensed data profile [22]. These anomalies occur due to several reasons and among them are: errors in the measurements caused by faulty sensor nodes, some noise gained by external factors, actual events because of the changes in the sensed environment, or malicious attacks launched by compromised sensor nodes. Anomaly detection as defined in [22] refers to the problem of finding patterns in data that do not match with the well-established and expected behavior.

#### 3.3.2. Motivations for Anomaly Detection in Wireless Sensor Networks

One of the most important motivations for anomaly detection in WSN is to provide data reliability and quality since sensor data can be corrupted and damaged due to many reasons such as reading errors, faulty sensors, or malicious attacks. Event reporting is another motivation for building anomaly detection since many WSNs have been used recently for monitoring different kinds of phenomena, for example, weather changes and fire detection [36,37]. The use of anomaly detection for event detection helps in detecting such a disaster or serious problem in its early stage and helps in making decisions accordingly. Furthermore, an event may be of interest for further analysis by scientists. Malicious sensor nodes that are compromised by some adversaries in WSNs are another strong motivation for building anomaly detectors. Such adversaries may gain access and control of some nodes and then start launching attacks that either can drain the limited resources of the network or inject false and corrupted data. Subsequently, this data may be used later by to make false decisions. Some works [9,38–41] have tackled this problem and use anomaly detection to detect such kinds of attacks so that a suitable action can be taken. A comprehensive survey of intrusion detection schemes in WSNs can be found in [42].

#### 3.3.3. Characteristics of Sensor Data

Sensing data are collected in the form of data streams which may be large volumes of real observations collected from the environment [43]. Some WSNs are designed only to collect one type of data such as temperature, light, humidity. This kind of data is called univariate data. Recent WSNs are designed to collect multiple types of data from the field simultaneously, which are called multivariate data. The nodes in these networks are usually equipped by more than one sensor to collect different types of data at the same time. In the multivariate data, each type of data is called an attribute or feature. A sensing data measurement is said to be anomalous if one or more of its attributes are anomalous [44]. With univariate data, the anomaly detection can be easily achieved by observing that the single data attribute is anomalous compared with the attributes of other data instances. However, anomaly detection in multivariate WSNs is challenging because the individual attributes may not show anomalous behavior but when are taken together they may display anomalous behavior [45]. Though analysis of multivariate data is computationally expensive, anomaly detection on multivariate data gives high accuracy if the relations between different attributes are carefully exploited [46,47]. Sensing data also has spatial and temporal correlations between sensor readings. Temporal correlation means that the readings collected at one time period are related to the readings collected at the previous time period. Spatial correlations means that the readings of nodes that are geographically close to each other are expected to be correlated [48]. According to [9], the spatial and temporal correlation between sensing data attributes help to specify the source of anomaly.

### 3.4. Challenges of Anomaly Detection in Wireless Sensor Networks

The main challenge of anomaly detection in WSN is how to achieve high detection effectiveness with minimum energy cost. In other words, the aim is to provide high detection effectiveness and high efficient resources utilization at the same time during the design of anomaly detection solution. Many anomaly detection solutions have been proposed in the literature for traditional networks systems. However, the nature of sensor data and the context of WSNs make these solutions unsuitable to be applied for WSNs. The challenges that should be considered during the design of suitable anomaly detection solution for WSNs are summarized in the following paragraphs:

*Computational and Storage Resource Limitation*: WSNs are made up of cheap sensors which are very resource constrained in terms of memory, and processing. The process of anomaly detection in WSN requires the utilization of the computational and storage resources for processing data in real time.

*Communication Overhead*: Some traditional anomaly detection solutions are built based on the centralized approach in which the data is collected from sensors and sent entirely to be processed by the cluster head or the base station. However, the cost of data transmission is several orders of magnitude higher than the cost of data processing [1]. In addition, the design of distributed online anomaly detection models also requires the communication between sensor nodes. As a result, the energy consumption is affected by the amount of communication overhead incurred by the distribution process.

*Dynamic Network Topology Change*: The mobility of nodes in some WSN applications and the communication failures increase the network topology change. This change negatively affects the validity of the normal reference model used by the anomaly detector.

*Network Heterogeneity*: Sometimes, the application of WSN needs to use different types of nodes or assign different jobs to different nodes. In addition, the current sensor nodes may be equipped with many sensors for measuring different environmental phenomenon at the same time. Another aspect of heterogeneity appears when the data collected by sensors obey to different data distributions which make the anomaly detection model, which was learned using one type of distributions, not capable to cope with such kind of heterogeneity.

*Dynamic Streaming Data*: The dynamic streaming nature of sensing data is another challenge. Generally, there is no prior knowledge available to build the normal distribution of sensing data (normal reference model). Even if this knowledge is available in a specific point of time, it is insufficient for the future because of the dynamic streaming that may change the nature of distribution over the time.

*Network Scalability*: Some WSNs applications expand over the time such that some nodes may be added to the network. As a result, the old normal reference model which was built for the network needs to be updated. The high false alarm rate resulting from the expansion poses a challenge to anomaly detection. Besides, the large amount of data produced due to network size expansion is also a challenge for real time detection [9].

*High Dimension Data*: Besides the possible increase of network size, the dimensionality of the collected data may also increase. The increase of data dimensions incurs a higher computational cost that drains the energy and memory of sensors. As the anomaly detection process depends on the data measurements, the increase of data dimension becomes a problem for efficiency aspect of anomaly detection.

### 3.5. Requirements of Anomaly Detection in Wireless Sensor Networks

Based on the challenges discussed in the previous section, the RODAC requirements are highlighted. These requirements intend to cover most of the challenges while the remaining requirements are left for further investigation.

*Data Dimensions and The Need for Dimension Reduction*: sensor data is categorized based on its dimension into univariate and multivariate data, according to the phenomenon's characteristics. In multivariate data, the samples may originate from different sensors of a specific node or from different nodes. It is clear that, transmission of multivariate data will increase the energy consumption in sensors because of the radio communication overhead involved for each variable. Therefore, data transmission between sensor nodes and central locations such as cluster heads and base station is the main reason for quick sensor energy consumption. According to [32], the transmission of one bit of data consumes the power needed to process thousands of bits in sensors. Besides, additional energy is required for processing large-scale and multivariate data [1,3,9,29]. As a result, multivariate data dimensionality reduction is a necessary task for reducing the energy consumption and hence prolonging sensor network lifetime.

*Operation Mode*: early anomaly detection models for WSNs [46,49–53] have not considered the online detection of data anomalies. Instead, detection was performed after time windows specified by the design of WSN application. Although offline detection models consume less energy, they require additional memory for storing data batches for the specific time window. Besides, data integrity could be affected due to the detection delay time. Therefore, online detection is preferable to minimize the delay time and ensure data integrity. An additional factor that affects the use of online detection mode is the cost of detection methods in terms of computations. The candidate method for online anomaly detection should be lightweight to cope with the resource limitations (energy, processing, storage, and bandwidth). The efficiency of any proposed anomaly detection solution is not affected only by the dimension of data but also by the computational complexity of methods used for detection. For example, it was reported that the one class support vector machine method incurred a computational complexity of *O*(*M*^3^) where *M* is the number of measurements in a specific time window [54]. Such complexity quickly depletes the limited sensor energy.

**Figure 4.**
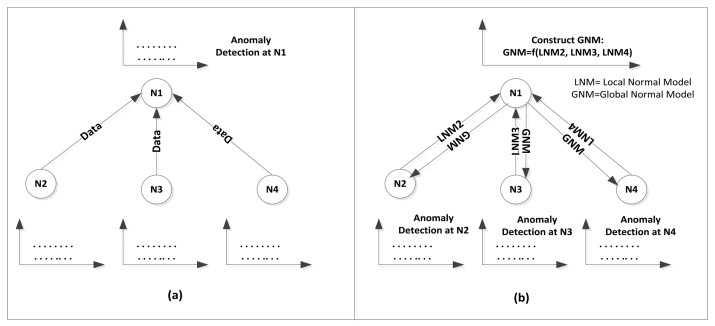
(**a**) Centralized *vs.* (**b**) distributed model structure for anomaly detection in wireless sensors networks (WSNs).

*Model Structure*: three structure types were adopted for existing anomaly detection models which are local, centralized, and distributed structure. In a local structure, the anomaly detector is implemented in the node scope with no collaboration between nodes in the network. In a centralized structure, the whole data is sent to a central location such as base station or cluster head where the anomaly detection process takes place. Finally, the distributed structure adopts collaboration between nodes for the detection process in which each node sends a summary of its data represented by its local normal reference model to cluster head for the construction of global normal reference model. The global reference model is then used by each node in the cluster for subsequent detection. Figure 4 shows an abstraction of the centralized and distributed structures.

It is clear that centralized models incur high communication overhead in transmitting the whole data for detection in the centralized location. As mentioned before, most of sensor energy is consumed in transmission rather than processing. Therefore, distributed detection is preferable in order to minimize the energy consumption. Meanwhile, the size of local normal reference model which is sent to cluster head as summary should be of small size to reduce the communication overhead. Another factor that should be considered for distributed structure is how often the local reference models should be sent to the cluster head and therefore how often the global reference model should be reconstructed from the local reference models.

*Adaptability with Dynamic Data Changes*: due to dynamic streaming of sensor data measurements, the model that represents the normal data behavior becomes rigid over time. Thus, updating the normal model is crucial for effective anomaly detection. A variety of updating mechanisms were proposed in the literature [55–57], but their adoption for WSN is governed by their compatibility with the resource restriction demands of sensors. Moreover, the required updating mechanism should be lightweight and efficient to meet the resource restriction demands of sensor devices.

*Data Correlation Exploitation*: sensor data measurements of close neighborhoods are characterized by high attribute, spatial and temporal data correlations. The attribute/feature correlation can be exploited to improve the efficiency of detection models through data dimension reduction. Temporal correlation means that the readings collected at one time period are related to the readings collected at the previous time period. Spatial correlations means that the readings of nodes that are geographically close to each other are expected to be correlated [48]. Exploiting such data spatial and temporal correlations altogether increase the effectiveness of anomaly detection.

## 4. Detection Method-Based Classification of Anomaly Detection Models in WSNs

In this section, we classify the existing anomaly detection models based on the detection method used to design the model into: statistical-based, nearest-neighbor-based, clustering-based, classification-based, and others. Although similar to the classification of [9], our classification is more comprehensive and cover the most recent published models. Besides, the evaluation and analysis of each approach in our classification consider the RODAC requirements of existing anomaly detection models as presented in previous section.

### 4.1. Statistical-Based Anomaly Detection Models for WSNs

The statistical-based anomaly detection models are the earliest models used for anomaly detection and first used for one dimensional data sets [21]. The essential principle of these models is to build a statistical normal model in the form of probability distribution which represent the distribution of the data in a reference model and evaluate each pattern with respect to that reference model. Any deviation from the reference model is considered as anomaly. More technically, if the probability of a pattern with respect to the statistical model is low, it is considered anomalous. Many statistical techniques have been used for anomaly detection in WSNs and categorized in [9,18] into parametric and non-parametric techniques. In the parametric category, it is assumed that the data is generated from a known distribution and then the parameters of distribution are easily estimated from this data. In the non-parametric category, the underlying data distribution is not known *a priori*. Instead, some estimation techniques such as histograms are used to estimate the underlying data distribution and therefore build the reference normal model that characterizes the behavior of data. Another categorization of the statistical techniques was presented in [29] and based on the structure of the network, hierarchical or flat WSN structure. The following paragraphs study existing statistical-based models for anomaly detection in WSNs.

*Palpanas et al.* [53]: a distributed deviation detection model in WSN was proposed to avoid the unnecessary communication overhead and computational cost. This model, which is based on a non-parametric statistical technique called kernel density estimator, was aimed at dealing with dynamic streaming data. The study has emulated the hierarchical structure by assigning each group of low capacity sensors to one of a limited number of more powerful sensors based on spatial proximity. This model was not evaluated experimentally and only described theoretically to show the tradeoff between detection effectiveness and efficiency. In addition, there is a dependency on a single threshold which does not suit multivariate data.

*Subramaniam et al.* [58]: this model is an enhancement of the kernel density estimator based model proposed in [53] by adapting a cluster based structure for WSN. A sliding window-based chain sample algorithm was used for online sensing approximation. Meanwhile, a probability-based mechanism was adopted to update the normal reference model regularly to meet the dynamics of data streaming in WSNs.

*Zhang et al.* [59]: five online and distributed statistical-based outlier detection techniques for WSNs named temporal outlier detection (TOD), spatial outlier detection (SOD), spatial predicted-data-based outlier detection (POD), temporal and spatial real-data-based outlier detection (TSOD), and spatial and temporal integrated outlier detection (STIOD) were proposed in this study. Each technique has each own drawbacks that are inherited from its design principles. It was reported that the TOD reduced the communication overhead but produced low accurate detection. On the other hand, SOD achieved high accuracy, but with high communication overhead. POD and STIOD have reduced the communication overhead, but still with low accuracy. The authors suggested that the TSOD is the better choice among those five techniques as it provides better detection accuracy of outliers locally at each node. However, the TSOD still incurred a high communication overhead due to the incorporation of spatial correlations and therefore affects the efficiency of the proposed techniques.

*Bettencourt et al.* [12]: this study presents two parametric statistical models for identifying outlying sensors in WSNs. These two models follow the Gaussian-based models in which the underlying distribution of data is normal. Both spatial and temporal correlations of sensor readings were exploited to help in detecting the source of outliers. In both models, a suitable threshold value was used to compare with the deviation of sensor data. If the deviation exceeds the predefined threshold, the sensor pattern is considered anomalous.

*Sheng et al.* [60]: in this work, a histogram of the sensing data is sent to the base station instead of data itself, which reduces the computation and communication cost. The base station is responsible for extracting the data distribution from the histogram. An estimating kernel function [53,61] is used to identify anomalies in the streaming data online. This approach is distributed as each node is responsible for detecting anomalies locally using the underlying data distribution estimated by the kernel density estimator from the streaming sensor data.

*Sharma et al.* [61]: in this study, the detection of three faults types which are short, noise, and constant reading faults were studied. The authors evaluated the performance of three different data faults detection methods that fall into three main models: rule-based, estimation-based, and learning-based. The methods worked well with high and medium intensity of short injected faults and with the high intensity of noise injected faults. However, these methods failed in most cases to detect long or constant injected faults and the low intensity short and noise injected faults. For real world datasets, it is reported that these methods performed generally well as these datasets experienced high intensity faults.

*Yao et al.* [62]: in this work, an online anomaly detection approach for sensor systems was proposed. The segmented sequence analysis (SSA) algorithm was used to construct a piecewise linear model of sensor data time series. In this approach, data anomalies are detected by comparing the constructed piecewise linear model of data collected in a fixed time period with a reference model using similarity metrics. If there is a significant difference, an anomaly alarm is flagged. This model can be considered as a distributed anomaly detection model that has two layers of detection. The first layer is the local detection at nodes and the second layer is in the cluster head. However, there is no feedback from the cluster head to the local nodes and this issue was left for future work.

*Miao et al.* [63]: in this work, a histogram-based online anomaly detection scheme was proposed for a hierarchical WSN. This scheme was claimed to overcome the drawbacks of existing histogram-based schemes which include the verification procedure that cost high computations and communication overhead. To solve this issue, the authors introduced a simple estimation approach that works online and in a distributed manner. However, some drawbacks still exist in this scheme such as parameter selection, and the inability to cope with unexpected changes of normal model behavior. Besides, like other histogram-based schemes, they are limited to univariate data and cannot support multivariate data and hence this limits the generality of the scheme.

Although statistical based anomaly detection models are commonly used in WSNs, they have some common limitations.

- Statistical models which rely on the underlying data distribution such as parametric techniques-based are not useful because in most WSN real life applications, there is no prior data distribution knowledge.
- Histograms do not rely on the underlying data distribution but they are only efficient for univariate data and cannot find the interactions between attributes in multivariate data.
- The threshold selection is application dependent and it is a difficult task, especially for the continuous dynamically changed environment.

### 4.2. Nearest-Neighbor-Based Anomaly Detection Models for WSNs

Nearest-neighbor-based models are a branch of data mining and machine learning community. They have been used for anomaly detection in computer networks with the assumption that normal patterns of data are always found in dense neighborhood while the anomalous ones are far from their neighbors [22]. The concept of these models is based on the use of similarity measures that measure the degree of data pattern being normal or anomalous, such as Euclidean distance measure.

*Branch et al.* [52]: in this study, an in-network outlier detection model was proposed based on the calculation of the distance similarity between data instances to find the global anomalies in WSN. In this model, each node applies the distance similarity to find the anomalies and broadcast its result to its direct and next hop neighbors. Other nodes perform this task until all nodes agree on a common decision about anomalous measurements. However, the broadcast communication leads to high energy consumption especially when dealing with large scale WSN.

*Zhuang and Chen* [64]: in this work, an in-network outlier cleaning and removal approach for WSNs was proposed. The wavelet-based approach was used for outlier correction and the neighboring Dynamic Time Warping (DTW) distance based similarity method was used for outlier detection and removal. Two outlier types were addressed by the proposed models which are the short simple outlier in zero hop and the long segmented outliers within two hops. The wavelet approximation method was adopted to correct the short, occasionally appeared outliers. Authors claimed that since the short outliers are of high frequency, they can be detected using the first few wavelets that represent the sensing series. Moreover, the use of wavelets helped in reducing the dimension of data and hence reduces the communication cost in the network. The dimension reduction concept by transforming the data to another space was also conducted using an adaptive PCA approach [65]. Meanwhile, the long segmented anomalies were addressed by the use of DTW distance-based method given that environmental data are spatially correlated. A similarity comparison with neighboring nodes is conducted within two hops to detect the long segmented outliers. The sensing series are not forwarded to the sink if the dissimilarity from neighboring nodes is greater than DTW threshold. The computational cost of the proposed approach and the dependency on the threshold reduced it suitability for online outlier detection which is an important issue especially for highly dynamic environments.

*Xie et al.* [66]: an unsupervised distance-based anomaly detection model was proposed for efficient anomaly detection in this study. The model incorporated the PCA for reducing the dimension of data variables before calculating the distance measure. It was claimed that the dimensionality could be reduced to one in any situation when validating with a static IBRL dataset. However, the model needs to be validated for dynamic datasets such as environmental ones to prove the dimension reduction claims. Without data dimension reduction, this model poses a high computational cost due to the calculation of the distances on multivariate datasets.

*Miao et al.* [67]: these authors investigated the problem of lazy learning of k-NN algorithms and the difficulty of using them for online detection in WSNs. In order to overcome the lazy learning issue, the authors proposed a new k-NN-based anomaly detection scheme based on hyper-grid intuition. It was reported that the new adoption reduced the computational cost significantly, which made the proposed scheme effective, robust and suitable for online detection. However, this scheme was only validated on a static and homogeneous dataset and needs to be validated on more dynamic datasets to show its ability for coping with dynamic changes.

Some limitations of nearest-neighbor-based models can be stated as follows:

- The computation of the distance between data patterns in multivariate datasets is very expensive.
- As a result on the first drawback, the scalability of these models is a major concern.

### 4.3. Clustering-Based Anomaly Detection Models for WSNs

Clustering models are among the most important data mining models which are used to group similar patterns with similar characteristics into clusters. A cluster is said to be anomalous if it is either smaller than or distant from other clusters in the dataset [9,27,68,69]. To determine the membership of a data pattern to a cluster, different similarity measures are used. Among these measures is the Euclidian distance measure. Clustering techniques have been used in WSNs to find the most efficient way for communication and processing of data [70].

*Rajasegarar et al.* [50]: in this study, a distributed anomaly detection model based on clustering and k-NN technique was proposed. Each sensor node collects the data and performs the clustering locally instead of sending the whole data to the base station or cluster head.

**Figure 5.**
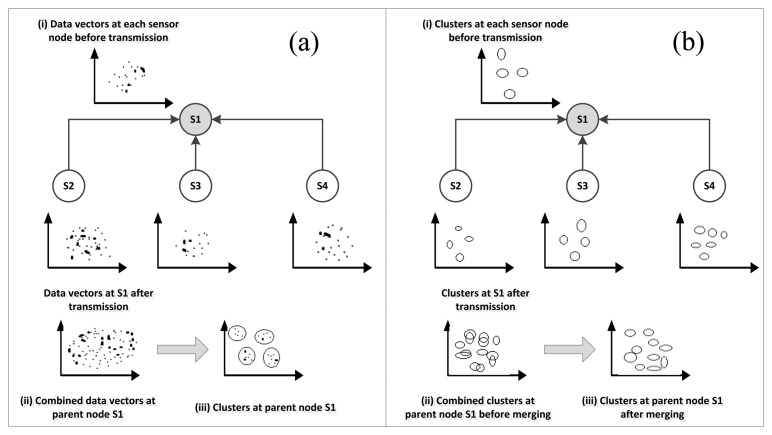
(**a**) Centralized detection model (baseline); (**b**) the distributed model [50].

Each node then builds its local normal reference model composed of cluster centroid and number of instances in the cluster and sends it to the cluster head where the global normal reference model is constructed from the local reference models sent by all nodes in the cluster. The global reference model is then sent back to the sensor nodes to be used for detecting potential anomalies in subsequent measurements. The cluster head upon receiving the cluster summaries from the nodes forwards them to the base station where a k-NN based technique is used to cluster normal clusters from anomalous ones. Figure 5 depicts the detection process in this model. The model was benchmarked against the baseline centralized model. It was reported that distributed model shows almost similar accuracy but with high communication overhead reduction compared to the centralized model.

*Rajasegarar et al.* [71]: the authors proposed a distributed approach for anomaly detection based on the ellipsoidal clustering method. The detection mechanism used in this approach was similar to the mechanism used in [50]. The authors first used the geometry of hyper-ellipsoids to model the normal behavior of sensor data measurements, and to provide a formal definition for anomalies based on the elliptical anomalies concept. The main challenge of the proposed approach according to the authors was how the elliptical anomalies can be detected accurately while consuming less energy. From that challenge, a distributed approach to anomaly detection was developed, which significantly reduces communication overhead in comparison to a centralized approach that communicates all data to a central node where anomaly detection is performed. It was shown that the distributed approach has the same accuracy as the centralized approach, while reducing the communication overhead significantly.

*Bezdec et al.* [72]: an elliptical summaries anomaly detection system (ESAD) was described in this study. Data measurements are collected at individual sensors and converted to elliptical summaries using the same method of [71]. The focal dissimilarity measure was used to build the matrix of focal distances from ellipses pairs and used as basis for assessing the tendency with iVAT images. To detect anomalies, the single linkage clustering algorithm was used to extracts clusters from dissimilarity data that groups normal and anomalous measurements in different clusters.

*Suthaharan et al.* [73]: in this approach, an ellipsoidal boundary of sensor observations was defined based on raw data transformation. This approach is different from the approach proposed in [71] in that the elliptical boundary of data measurements is derived from transformed data based on a Gaussian distribution instead of actual data. The authors claimed the simplicity and effectiveness of this approach in terms of enhancing detection accuracy and reducing energy consumption. Furthermore, the proposed approach is different from the standard approach of [56] in that it does not rely on a threshold that depends on a certain percentage of data measurments to be included in the ellipsoid. Instead, a suitable covariance matrix is derived based on the distance between data points. The problem of this approach is the high computational complexity that results from the data transformation and distance calculations to find the radius. This complexity affects the suitability of the approach for real time applications.

**Figure 6.**
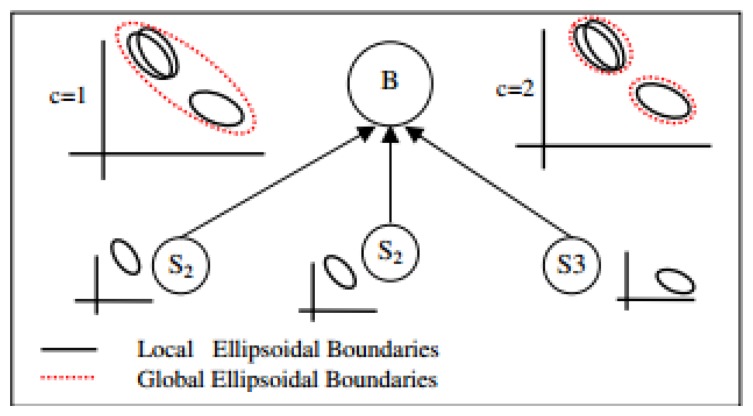
Clustering ellipsoids for anomaly detection in WSNs [69].

*Moshtaghi et al.* [54,69,74]: the authors in [69] used ellipsoidal clustering for anomaly detection in heterogeneous WSNs in which more than one type of data were collected. The difference of this approach from the ellipsoidal clustering approach of [71] was the ability to deal with heterogeneous applications that have different data distributions at the same time. The procedure of detecting elliptical anomalies in this approach is depicted in Figure 6 and can be summarized as follows:

- At each sensor node, the local ellipse is calculated from the data measurements collected by that node for specific period of time. The ellipse parameters are then sent to base station.
- At the base station, the similarity between each pair of ellipses received from all nodes is calculated and the number of clusters for each ellipse is estimated.
- The ellipses are then clustered into a set of merged clusters and the parameters of merged ellipses are transmitted to each sensor node.
- At each sensor node, the merged ellipses are utilized to detect global anomalies by flagging any measurement that fall outside the merged ellipses as anomalous.

In [54,74], incremental versions of the ellipsoidal clustering models of [69] were proposed to cope with the dynamic changes of the monitored environments. It was claimed that these incremental models can successfully detect anomalies in an effective manner with high detection accuracy.

The use of clustering models helps in reducing the communication cost by clustering the data locally. Besides, there is no need to have prior knowledge of data since clustering is fully unsupervised.

Three main drawbacks are identified and need more consideration when using clustering techniques for anomaly detection in WSNs, which are:

- Dependency on the choice of cluster width in some clustering techniques makes them not suitable for WSN applications.
- Clustering is very computationally expensive with multivariate data because the calculation of the distance measures among all data patterns has high computational cost that make them unsuitable for limited resource devices such as sensors.
- Clustering techniques cannot cope with continuous changes of data streams over time so the normal reference model will be out of date by the time they are used. Although, some recent clustering-based models [54,74] have tackled this issue via incremental learning methods, the computational cost for such methods is too high to be affordable by constrained resource devices.

### 4.4. Classification-Based Anomaly Detection Models for WSNs

Classification models are important models of machine learning and data mining community in which the classifier is trained using known training data patterns and used after that to classify the unknown patterns into one or more types. Supervised classification on multiclass data are not used in WSN due to difficulties in obtaining labeled sensor data. Meanwhile, one class supervised classifiers are the type of classifiers that learn the normal patterns and consider any pattern falls outside the boundary of normal as anomaly. One class supervised classifiers are more suitable for designing anomaly detection models in WSN, because the normal patterns can be gathered to train the classifier.

*Janakiram et al.* [46], *Hill et al.* [75]*and Elnahrawy and Nath* [76]: three types of Bayesian networks were used for anomaly detection in WSNs which are the Naïve Bayesian Network (NB) [76], Belief Bayesian Network (BBN) [46], and Dynamic Bayesian Network (DBN) [75]. In [76], the problem of learning from the spatial and temporal correlation in sensor data was mapped to the problem of learning the NB parameters and then making the interference. Meanwhile, in [46] the conditional dependencies among data attributes were exploited and claimed to increase the detection accuracy. The authors in [75] proposed DBN to cope with the changes caused by dynamic changes in WSN topology over the time. This technique detects anomalies by computing the posterior probability of recent data patterns in a specific period of time called sliding window.

**Figure 7.**
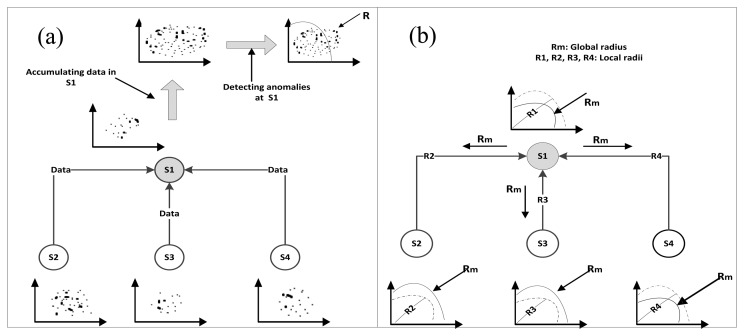
(**a**) Centralized *vs.* (**b**) distributed anomaly detection using QSSVM approach [51].

*Rajasegarar et al.* [51]: in this study, a Quarter Sphere Support Vector Machines (QSSVM)-based distributed anomaly detection model was proposed to identify the anomalous observations in the data. The model structure is similar to the structure of the model proposed by the same authors in [50]. The procedure of applying this technique is shown in Figure 7 and summarized as follows:

- Each node runs the QSSVM on its local data and finds the local anomalies and the local radius *Rj* of the QSSVM.
- Each node sends the local radius *Rj* to its parent.
- The parent collects the radii sent by its children and combines them with its own radius and form the global radius *Rm*.
- Parent node sends the global radius to its children to detect anomalies in their data using this radius.

The model was evaluated by comparing the proposed distributed model with the centralized model. The results showed an improvement in terms of accuracy and communication overhead using the distributed model which helps to prolong the lifespan of WSN. Figure 7 provides an example of this model and clearly shows how the distribution was implemented. The main drawback of this model is the offline training of QSSVM since the local radius in nodes is calculated on data collected for a period of time, so this model is not suitable for the streaming demands of WSNs. In addition, the computational complexity of the SVM introduces a question about the energy consumption which is the main concern in designing any solution for WSNs. Furthermore, the selection of SVM parameters affects the flexibility of the model for dynamic environmental changes.

*Rajasegarar et al.* [77]: authors proposed a hyper ellipsoidal SVM-based anomaly detection model to overcome the drawbacks of the previous model based on a quarter sphere SVM proposed in [51] in terms of inflexibility of selecting parameters and computational complexity. Although higher detection accuracy was achieved using the new SVM based model, it was not suitable with the distributed structure because it increased the communication overhead according to the authors.

*Zhang et al.* [41,78–80]: an online anomaly detection model based on the one-class QSSVM was proposed in [78]. This model used the same detection method previously used in [51]. The difference between the two models is in the network structure. In [51], a hierarchical based WSN was adopted in which the network is divided into clusters and the construction of the global normal reference model is built in cluster heads, whereas in [78], a flat-based WSN structure was adopted in which a group of nodes that form a close neighborhood have a group manager node that builds the global normal reference model. The communication overhead incurred by nodes in the group is higher than the communication overhead incurred by transmissions in a cluster which gives the model of [51] an advantage over the model in [78]. However, the model in [78] operates in an online manner which offers it an advantage for real time applications.

An adaptive and online one-class SVM based anomaly detection model was proposed in [41,79] based on the model proposed in [78]. Similar to the models in [51,77,78], the one-class QSSVM technique was also adopted for this model. The difference here is the online training of the SVM in which the normal reference model represented by the radius Rj in each node is sequentially updated in different ways. Three different mechanisms of updating the normal reference model were investigated and studied, namely instant outlier detection (IOD), fixed-size time window-based outlier detection (FTWOD), and the adaptive outlier detection (AOD). In IOD, the normal reference model is calculated for each training instance, whereas in FTWOD, the normal reference model is updated at a fixed size window. These two update mechanisms incur high power consumption due to the broadcast of the normal reference model by the nodes to the parent node each time an instance is observed (IOD) or in each specific period of time (FTWOD). Therefore, AOD was designed to overcome the drawbacks of IOD and FTWOD so that the update process is carried out when there is an impact on the previous normal reference model by a measurement. So, this mechanism depends on the previous decision results. Although this model adopts online processing and adaptability to data changes, it still has some drawbacks. The use of SVM requires setting up some important parameters of which their impact on the detection was totally ignored. The same situation goes for the type of kernel function which may vary from one WSN application to another.

More recently, the authors in [80] proposed two distributed and online outlier detection techniques based on the hyper-ellipsoid one-class SVM. To increase detection rates and reduce false alarms, a data attributes correlation was considered. In addition, for further enhancement of detection accuracy, the same updating strategies of the normal reference model used in [79] were also utilized for this model.

*Chatzigiannakis and Papavassiliou* [49]: detecting anomalies and identifying faulty nodes using PCA was introduced in this work. PCA was applied simultaneously on multiple metrics received from sensor nodes. The high level anomaly detection model representation showed two types of analysis; offline analysis and real time analysis. In the offline analysis, enough normal data samples were collected from sensor nodes and fed to a PCA engine. The result of applying PCA is the selection of subset of PCs that retains the maximum amount of variance of sensor data. The selected PCs were then fed into the real time analysis unit in order to compare them with the real time data instances using a subspace method. Finally, based on a predefined threshold, the data instance is classified as normal or anomalous and followed by the determination of the anomalous node. The use of the subspace distance method in the online analysis unit is the major drawback of this model as it incurs high and prohibitive computations.

*Siripanadorn et al.* [81]: a combination of discrete wavelet transform (DWT) and self-organizing map neural networks (SOM) was developed to detect data anomalies in WSNs. In this study, data faults were considered as anomalies. The data measurements were first encoded at each node using DWT and then sent to the base station where the SOM was applied on a batch of wavelet coefficients. The sensitivity of the SOM to the possible noise in the training data makes it difficult to differentiate the source of anomaly. Moreover, as this model adopts the centralized structure, high energy consumption is occurred due to high transmission of data to the base station. In addition, as DWT is used to encode data locally, it involves high computation cost.

*Takianngam and Usaha* [82]: a combination of DWT and one-class support vector machines (OCSVM) was proposed. In this model, DWT was used for encoding the data measurements at each node like in [81]. The encoded measurements are then examined for anomalies by the OCSVM at the base station. This model has also the same limitations of the model in [81] in terms of high computational cost of DWT in the nodes and the high communication overhead caused by transmitting the whole data to the base station.

*Dereszynski et al.* [83]: a method for real-time data quality control that utilizes the data spatial and temporal correlations to distinguish faulty sensor observations from valid observations was presented. The adaptability with environmental changes was achieved by using a Bayesian network structure that captures spatial relationships between sensors in the close neighborhood. Furthermore, the temporal correlations were also incorporated by extending the structure to a dynamic Bayesian network. This model was claimed to be able to detect faulty observations and even predict the missing or corrupted readings correctly. A dataset samples from the Sensorscope project were used to evaluate the performance of the model. Experimental results of this model showed that considering spatial and temporal correlations of sensor observations enhanced the result of detecting faulty observations compared to the models that consider either spatial or temporal correlations individually.

*Bahrepour et al.* [37]: proposed an in-network decentralized approach for event detection in WSNs based on machine learning techniques. This approach adopted decision trees for distributed event detection and a reputation-based voting method for detection results aggregation over the sensor nodes in order to reach a consensus decision. The authors stated that the use of decision tress achieves highly accurate results in terms of detection accuracy with simplicity. Although the time complexity of the machine learning techniques used to design this approach was thoroughly discussed, the communication overhead which is the main player of energy consumption was not investigated.

*Shahid and Naqvi* [84] and *Shahid et al.* [85]: An energy efficient outlier detection approach that utilizes temporal and attribute correlations in WSNs was introduced based on the quarter sphere SVM (QSSVM) technique. It was reported that this approach increased outlier and event detection rates compared to previous models that use the same technique. Although, the communication overhead was reduced by exploiting temporal and attributes correlation, the computational complexity in individual sensors was increased.

*Shahid et al.* [86]: since the communication overhead incurred by the models proposed in [84] and [85] was significantly high, the authors in [86] present three partially online strategies to detect outliers and events based on the STA-QSSVM proposed in [85].The authors claimed that the adoption of the new strategies lead to a significant reduction in communication cost and comparable performance. Furthermore, it was claimed that these strategies are comparable to the STA-QSSVM in the existence of a higher fraction of outliers and events in the data set.

*Shahid et al.* [87]: in this work, various formulations of One-Class SVM such as hyper-plane, hyper-sphere, quarter-sphere and hyper-ellipsoidal were analyzed. A number of anomaly detection models for WSN that were designed based on these formulations were analyzed with regards to some characteristics, including the type of input data, the exploitation of spatial, temporal and feature correlations, the setup of SVM thresholds, outlier types, the determination of anomaly type, outlier degree, the effects of dynamic topology change, the dynamic data distribution, and the network heterogeneity. The scope of the investigation was limited to the study of improvement and feasibility of the proposed techniques for detecting data anomalies in harsh environments. The result of the analysis revealed that the quarter-sphere formulations are the most feasible for outlier detection in WSNs due to their low computational cost and communication overhead which leads to high efficiency. Hyper-plane- and hypersphere-based techniques have expensive computational costs and cannot cope with different data distributions. Hyper-ellipsoid formulations incur high communication overhead with a computational cost lower than that of quarter sphere techniques.

*Rassam et al.* [88]: proposed a new one-class principal component classifier (OCPCC)-based anomaly detection model to detect anomalies in an online manner. It was claimed that the proposed model achieved high detection effectiveness compared to the one-class SVM model. However, the computational complexity produced by the calculation of principal components online and the clustering method used for calculating the detection threshold incurred high energy consumption that affects the lifetime of the network.

*O'Reilly et al.* [89]: the authors of this work proposed an adaptive algorithm to adjust the anomaly rate parameter represented by a model parameter of a one-class quarter-sphere SVM for anomaly detection in WSNs. The proposed model was aimed at improving the detection performance and minimizing the error rate when operating in an online manner. It was stated that the anomaly rate parameter selection is important to obtain optimal detection performance. The authors concluded that the proposed adaptive model improved the detection effectiveness compared to the static model when the ratio of anomalies varies in the dataset. However, the parameter tracking algorithm increased the computational cost of the proposed adaptive model compared to the static model.

*Curiac et al.* [90]: in this work, an ensemble based classification system which is composed of five diverse binary classifiers was proposed for anomaly detection in sensor measurements. These classifiers are the average based classifier, autoregressive linear predictor based classifier, neural network based classifier, neural network autoregressive predictor based classifier, and adaptive neuro-fuzzy inference system (ANFIS)-based classifier. Two kinds of input data which included measurement time series collected by the sensor under investigation and the measurements gathered from neighboring sensors were used as input for the ensemble classifier system which was implemented in the base station. The authors stated that the ensemble system assured the diversity of classifiers to build an efficient decision making system. However, the main drawback of the proposed systems is the high communication overhead produced by sending all the time series data of the sensor and its neighbors to the base station.

Some limitations of classification based models are summarized as follows:

- Classification techniques like SVM are computationally expensive and hence quickly consume the sensors' energy. These techniques are inefficient for the design of online detection models which are favorable for some WSN applications.
- Some of the proposed models have inefficiently dealt with highly dynamic data streaming. Although some adaptive classification-based models such as [41] were introduced, these models incurred prohibited computational cost that drain the limited energy of sensors quickly.
- Bayesian networks are good in exploiting the correlations and dependencies in sensor reading and data attributes but they cannot scale well with a high number of variables such as those of multivariate datasets.
- Some classification techniques, especially SVM, are parameter dependent. This dependency limits the generality of the solution to only some specific applications and further raises the need for human intervention.

### 4.5. Other Anomaly Detection Models for WSNs

Other anomaly detection models were proposed using different techniques either from the field of machine learning or statistics. In [91], an anomaly intrusion detection model was proposed using a cumulative sum (CUSUM) algorithm to detect different types of attacks as anomalies. This algorithm was claimed to be a very lightweight and powerful in detecting changes in sensor data so that it fits the restrictive demands of WSNs. The authors stated that the complexity of their algorithm is very low compared to rule-based models in terms of computation and storage.

By considering malicious attacks and events as special cases of anomalies, a variety of techniques were proposed for malicious nodes and intrusion detection such as those described in [14,15,24,38,39,92–107]. Other models such as those of [36,37,108] were proposed for event detection. These models belong to different disciplines, such as statistical, rule-based, data mining and computational intelligence, and game theory.

## 5. Analysis of Current Anomaly Detection Models

The design of any anomaly detection approach for WSNs should consider two main issues which are the effectiveness in detecting anomalies and efficiency in utilizing the network resources.

Through the review of several anomaly detection models for WSNs in Section 4, it was found that the common limitations of these models can be categorized under inefficiency and ineffectiveness issues. The dimensions of the sensor measurements have an impact on the anomaly detection efficiency, which leads to energy consumption issues. According to [32], transmitting one bit consumes the same energy needed for processing thousands of bits inside the sensor. Computational costs also play a role in consuming the energy of sensors, especially when the dimensionality is high. Generally, dimensionality reduction is important in most data mining applications because it is used to reduce the dimensions of the problem, decrease the noise, and improve the speed of the model by eliminating the unnecessary features [109]. In WSNs, the highly dimensional data collected in some applications make the task of anomaly detection difficult because the detector needs to learn the features of highly dimensional data with very restricted resources. Besides, as the distributed structure is preferred for the design of anomaly detection models, the communication overhead should be also considered. As a result, there is a need for dimensionality reduction which helps sensors sustain their energy and prolong network lifetime. Unfortunately, very few anomaly detection models have adopted the dimensionality reduction prior to the detection process [66,81,82,88]. Moreover, the computational cost of the dimensionality reduction techniques used in these models was high, and these models do not satisfy other elements of the RODAC requirements.

As mentioned, early anomaly detection models for WSNs have not considered the online detection of data anomalies [50–52,76]. Instead, detection was performed after time windows specified by the design of the WSN application. Although offline detection models consume less energy, they require additional memory for storing data batches for the specific time window. Besides, data integrity could be affected due to the detection delay time. Therefore, online detection is preferable to minimize the delay time and ensure the data integrity. Many recent anomaly detection models adopt the online detection mode [54,63,67,78–80,88]. However, most of these models pose high computational costs inherent to the design of their detection methods. For this reason, anomaly detection methods should be carefully designed to ensure they meet the efficiency requirements and demands of WSN applications.

By considering the network structure adopted for anomaly detection models, it is clear that centralized models incur high communication overhead in transmitting the whole data for detection in the centralized location. The fact that most sensor energy is consumed in transmission rather than processing makes distributed detection preferable in order to minimize the energy consumption. However, the size of local normal reference models communicated with cluster head or master nodes should be small to reduce the communication overhead. Another factor that should be considered for distributed structures is how often the local reference models should be sent to the cluster head and therefore how often the global reference model should be reconstructed from the local reference models.

Due to the dynamic streaming of sensor data measurements, the model that represents the normal behavior of data becomes rigid with time. Thus, updating the normal model is crucial for effective anomaly detection. Few anomaly detection models have considered the adaptability issue [54,63,67,79,80]; though, these models pose high computational cost in tracking data dynamic changes. Additionally, in some cases, these models misclassified the normal measurements and hence increase false positive rates. Consequently, more efficient and effective adaptive anomaly detection methods are required.

The attribute/feature correlation can be exploited to improve the efficiency of detection models through data dimension reduction. Few detection models have exploited spatial and temporal correlations of sensor readings [46,59,79,80,85,86,88]. However, incorporating feature correlations with spatial or temporal correlations was not performed before. Such correlation is expected to enhance detection efficiency and effectiveness at the same time.

### 5.1. Evaluation of Current Anomaly Detection Models Based on the RODAC Criteria

Based on the analysis of the RODAC components in previous subsection, current anomaly detection approaches are evaluated with regards to the RODAC requirements. The evaluation is presented in Table 1. Some conclusions may be drawn from the evaluation presented in Table 1 and are summarized in the following paragraphs.

In terms of data dimension, univariate data was considered by some detection model whereas multivariate data was utilized by most detection models. Most recent WSN applications deal with multivariate data since sensors are equipped with multiple sensing units for different variables. Dealing with multivariate data leads to high energy consumption for processing and transmission. Therefore, feature correlations can be exploited to reduce the dimensions of multivariate data. Unfortunately, most of detection models that deal with multivariate data have not considered dimension reduction before the detection process. Only two anomaly detection models considered the dimensionality reduction process using PCA before detecting anomalies in multivariate sensor data measurements [66,88]. However, the high computational cost of the dimension reduction methods in those two models is the main drawback.

A number of reviewed models adopted the online detection mode whereas the majority of them were designed to process in offline manners. Online detection is necessary for some applications that operate in real time. Nonetheless, the high computational cost of the detection methods affects the practicality of the models that adopted online detection. Therefore, lightweight detection methods should be designed for real time applications.

Few existing models adopted the local or centralized structure whereas most of models were designed based on distributed structures. Several drawbacks were reported for the centralized structure. Although the availability of the whole data in the central location may increase the detection rate, it produces high communication overhead which is the main reason behind quick sensor energy depletion. Existing distributed models on the other hand have their own drawbacks that include the high computational cost of the detection methods. In addition, the size of the transmitted normal reference model summary also affects energy consumption. It is also shown that very few models have considered the adaptability with dynamic data changes.

Despite the fact some models [41,67,74] considered the updating of the normal reference model, their adaptability incurred high computational cost and parameter tuning issues. Besides, these models consider the update of the model based on time windows that may result in high update delay issues. Finally, few models have considered the spatial and temporal correlation that exist in sensor data measurements [41,46,79,84,86].

Few anomaly detection models [12,41,79,80,85,86] satisfy all RODAC requirements as highlighted in Table 1. However, these models still do not represent optimal anomaly detection solutions for WSNs for some reasons. The models proposed in [12] are parametric-based which requires the use of a suitable threshold to measure the amount of deviation of the sensor measurements. In fact, determining the suitable threshold for all applications or for one application in dynamic environments is not a trivial task. The classification-based models proposed in [41,79,80,85,86] were designed using the one class quarter sphere SVM. The common limitations of the three models include the high computational complexity that makes them not practical for real time WSN applications. Moreover, the use of SVM requires the tuning of some important parameters which requires human intervention and hence affects the flexibility of these models.

In the end, none of the proposed models listed in Table 1 satisfies all the required RODAC components for efficient and effective anomaly detection. The aim is to consider all these components together without incurring additional computational cost or communication overhead and hence minimize energy consumption. The following section summarizes the general limitations of the current anomaly detection models explored in previous sections and suggests some opportunities to be considered for future research.

## 6. General Limitations of Current Anomaly Detection Models and Open Issues

### 6.1. The Limitations of Currents Anomaly Detection Models

After exploring the various anomaly detection models, a summary of their limitations is given in the following points:

- Most current WSN applications are designed to deal with multivariate data whereas some existing models dealt with univariate data. In multivariate data, the features together may pose an anomaly whereas individually they may not. Even though some models dealt with multivariate data, they have not considered data dimension reduction.
- Although recent proposed models were designed to operate online, the computation cost of their detection methods is a major drawback that causes an increase of energy consumption.
- Most current models adopt the distributed structure for anomaly detection. Nonetheless, these models have some drawbacks related to the size of reference model that should be communicated between nodes. Besides, most existing distributed models have not clarified the method of combining the local normal models in a global normal model. Finally, these models have not determined the suitable threshold for updating the global normal model because of data dynamic changes.
- The adaptability with dynamic data changes that was incorporated in some recent detection models produced additional computational costs that affect their suitability for real time detection and update.
- Many proposed anomaly detection models ignore a special feature of WSN data which is its spatial and temporal correlations. This feature helps in improving detection accuracy. Additionally, attribute/feature correlations which show the dependencies among features were ignored by most of the existing works. Exploiting such feature correlations through feature reduction helps sustain sensors' energy.
- Some of models reviewed, especially statistical models and classification-based models suffered from parameter selection issues. Some parameters need to be tuned for the detection model prior to the detection process. The performance of some classification methods such as SVM is changed significantly by the changes of some user parameters. In real WSN applications, it is not easy to determine a suitable parameter value for each application. In addition, it is not suitable to assume fixed values if the aim is to consider the dynamic change of the WSNs data.

### 6.2. Open Issues

This section suggests some open research issues based on the study of existing models as in the following points:

*Dimensionality reduction*: there is a need for reducing the dimensionality of multivariate data in order to reduce the high energy consumption. Very few dimension reduction methods were used prior to the anomaly detection process. However, the computational complexity of these methods affects the detection efficiency as it incurs additional energy consumption. Therefore, more lightweight dimension reduction methods are needed. Some methods that share the same principle with PCA-based methods such as ICA and wavelets are potential alternatives.

*Online detection*: to meet the demands of some real time WSN applications, it is preferable for the detection process to be online. Although many existing models were claimed to be suitable for online detection, they incur high energy consumption caused by their adopted detection methods. Therefore, more lightweight detection methods are required. The one class supervised learning methods proved to be suitable choices for detection anomalous data measurements in sensor networks such as One Class SVM, One Class PCC. These methods transfer the problem to another space and solve the problem in linear form. The problems of these types of classifiers are the need for parameter tunings and the fact that threshold determination is required.

*Distributed structure*: to reduce the communication overhead caused by sending all data in centralized models, the distributed design of anomaly detection models is recommended. Although some distributed models were proposed, they still have some issues that should be overcome such as the length of the reference model that is communicated in the network and the need for updating this reference model based on the changes in this structure.

*Adaptive Detection*: due to dynamic streaming nature of sensor data, an update of the detection model is required in order to minimize false positives and improve detection accuracy. The few proposed adaptive strategies suffer from high computational complexity that affects their suitability for online detection. Therefore, modifying these strategies or proposing new lightweight ones is required.

*Feature/Attribute correlations*: temporal and spatial correlations of sensor data should be exploited in order to improve detection effectiveness and efficiency at the same time. These correlations should be combined together with feature/attribute correlations to improve the effectiveness and efficiency together.

*Parameter tuning*: to reduce human intervention, the design of anomaly detection model should avoid the manual setup of parameters. The design for automatic parameters tuning methods is preferred to reduce the human intervention.

## 7. Conclusions

Detecting anomalies in sensor readings efficiently and effectively is an important task to ensure the quality of collected sensor data for making right decisions. A variety of anomaly detection models were proposed in the literature; nonetheless, most of them suffer from low detection effectiveness or high energy consumption. In this survey, we studied the challenges that face the design of an efficient and effective anomaly detection model for WSNs and stated the requirements (RODAC components) that should be satisfied to design such models. These requirements include the need for data dimension reduction, online detection, distributed detection, adaptive detection and the exploitation of spatial/temporal correlations. A comprehensive review of the state-of-the-art detection models was proposed that categorized them based on detection techniques into statistical-based, clustering-based, classification-based, and nearest-neighbor-based. In each category, a brief description was given for each model and the overall limitations of the category were discussed. A comparison and analysis of all models from the four categories was conducted to study their satisfaction to the requirements stated in the RODAC components. The analysis reveals that none of the current models satisfies the full set of RODAC components. Therefore, a design of new models or enhancing the current models to consider these requirements altogether is required. The paper concludes by the providing the general limitations of current anomaly detection models and suggesting some directions for further research.

## Figures and Tables

**Table 1.** Evaluation of current anomaly detection models in WSN.

**Class**	**Approach**	**Data Dimension**		**Model Structure**			**Operation Mode**		**Adaptability with Changes**		**Correlation Exploited**	
		**Univariate**	**Multivariate**	**Local**	**Centralized**	**Distributed**	**Offline**	**Online**	**Adaptive**	**Not Adaptive**	**Spatial**	**Temporal**
**NN-based**	[52]		✓			✓	✓			✓		
	[64]	-	-			✓	✓			✓		
	[66]		✓			✓	✓			✓		
	[67]		✓			✓		✓	✓ *			
**Statistical-based**	[53]	-	-			✓	✓			✓		
	[61]	-	-			✓	✓			✓		
	[45]	✓		✓			✓			✓	✓	✓
	[12]		✓			✓		✓	✓ *		✓	✓
	[60]	✓			✓		✓			✓		
	[47]	✓	✓		✓		✓			✓		
	[62]	✓				✓ *		✓ *		✓		
	[63]	✓				✓		✓	✓			
**Clustering-based**	[50]		✓			✓	✓			✓		
	[71]		✓			✓	✓			✓		✓
	[72]		✓	✓			✓			✓		
	[69]		✓			✓	✓			✓		
	[74]		✓			✓	✓			✓		
	[54]		✓	✓				✓	✓ *			
**Classification-based**	[46]		✓			✓	✓			✓	✓	✓
	[75]	✓			✓		✓			✓		
	[76]	✓				✓		✓	✓		✓	✓
	[51]		✓			✓	✓			✓		
	[77]		✓		✓ *	✓ *	✓			✓		
	[78]		✓	✓				✓		✓	✓	
	[41]		✓			✓		✓	✓		✓	✓
	[79]		✓			✓		✓	✓		✓	✓
	[80]		✓			✓		✓	✓		✓	✓
	[49]		✓		✓		✓			✓		
	[81]	✓	✓		✓		✓			✓		
	[82]	✓	✓		✓		✓			✓		
	[83]	✓			✓			✓	✓		✓	✓
	[37]		✓			✓	✓			✓		
	[84]		✓	✓				✓		✓		✓
	[86]		✓			✓		✓	✓ *		✓	✓
	[85]		✓			✓		✓	✓ *		✓	✓
	[88]		✓	✓				✓			✓	✓
	[89]	-	-			✓		✓	✓			
	[87]	-	-	-	-	-	-	-	-	-	-	-
	[90]	✓			✓			✓		✓		

(*) means partially satisfy; (-) means not applicable.
